# Supplementary material for: Visual Motor Reaction Times Predict Receptive and Expressive Language Development in Early School-Age Children
Source: Brain Sci. 2023 Jun 19;13(6):965. doi: 10.3390/brainsci13060965 (PMC10295862; doi:10.3390/brainsci13060965)
Supplement: Supplementary file 1 [file brainsci-13-00965-s001.zip › brainsci-2448657-supplementary.pdf]

Supplementary Material

Supplementary Table S1 Post Hoc Comparisons of sex-related differences in PPVT, EVT and RAN

	**Gender**	**Prior Odds**	**Posterior Odds**	$BF_{10,U}$	**error %**
			**a. PPVT**		
Male	Female	1.000	0.830	0.830	0.010
			**b. EVT**		
Male	Female	1.000	0.265	0.265	0.011
			**c. RAN**		
Male	Female	1.000	0.265	0.265	0.011

Note. The posterior odds have been corrected for multiple testing by fixing the prior probability to 0.5 that the null hypothesis holds across all comparisons. Individual comparisons are based on the default t-test with a Cauchy (0, r = 1/sqrt (2)) prior. The "U" in the Bayes factor denotes that it is uncorrected.

Supplementary Table S2 Bayesian Pearson Correlations (5-6 group)

Variable		RCPM	AS	VS	AVS	EHC	PPVT	EVT	RAN
1. RCPM	Pearson's r	—							
	BF_{10}	—							
2. AS	Pearson's r	0.078	—						
	BF_{10}	0.312	—						
3. VS	Pearson's r	0.102	0.540	—					
	BF_{10}	0.321	3.033	—					
4. AVS	Pearson's r	0.195	0.849 ***	0.580	—				
	BF_{10}	0.389	1469.533	4.702	—				
5. EHC	Pearson's r	-0.434	0.201	0.061	0.375	—			
	BF_{10}	1.219	0.395	0.307	0.829	—			
6. PPVT	Pearson's r	0.207	0.184	-0.046	0.329	0.112	—		
	BF_{10}	0.403	0.378	0.304	0.649	0.326	—		
7. EVT	Pearson's r	0.445	-0.284	-0.219	-0.208	-0.486	0.647 *	—	
	BF_{10}	1.323	0.528	0.417	0.403	1.837	11.312	—	
8. RAN	Pearson's r	0.014	0.128	0.054	0.235	0.639 *	-0.002	-0.371	—
	BF_{10}	0.300	0.335	0.306	0.438	10.120	0.300	0.812	—

Note. Age = age in numbers; RCPM = nonverbal IQ of Raven; AS = MRTs of auditory stimuli; VS = MRTs of visual stimuli; AVS = MRTs of audiovisual stimuli; EHC = SLURP visual motor skills; PPVT= Peabody picture vocabulary test; EVT= expressive vocabulary test; RAN = rapid automatized task. * $BF_{10} > 10$, ** $BF_{10} > 30$, *** $BF_{10} > 100$.*

Supplementary Table S3 Bayesian Pearson Correlations (7-8 group)

Variable		RCPM	AS	VS	AVS	EHC	PPVT	EVT	RAN
1. RCPM	Pearson's r	—							
	BF_{10}	—							
2. AS	Pearson's r	-0.212	—						
	BF_{10}	0.404	—						
3. VS	Pearson's r	-0.458	0.745 ***	—					
	BF_{10}	1.904	205.786	—					
4. AVS	Pearson's r	-0.514	0.751 ***	0.856 ***	—				
	BF_{10}	3.425	240.997	12791.937	—				
5. EHC	Pearson's r	0.137	-0.098	-0.169	-0.148	—			
	BF_{10}	0.323	0.300	0.351	0.332	—			
6. PPVT	Pearson's r	0.479	-0.213	-0.306	-0.373	-0.159	—		
	BF_{10}	2.348	0.405	0.621	0.945	0.342	—		
7. EVT	Pearson's r	0.622 *	-0.236	-0.358	-0.420	0.145	0.660 *	—	
	BF_{10}	15.206	0.444	0.850	1.362	0.329	29.668	—	
8. RAN	Pearson's r	-0.078	0.210	0.026	0.052	0.052	-0.206	-0.206	—
	BF_{10}	0.291	0.401	0.278	0.283	0.283	0.395	0.394	—

Note. Age = age in numbers; RCPM = nonverbal IQ of Raven; AS = MRTs of auditory stimuli; VS = MRTs of visual stimuli; AVS = MRTs of audiovisual stimuli; EHC = SLURP visual motor skills; PPVT= Peabody picture vocabulary test; EVT= expressive vocabulary test; RAN = rapid automatized task. * $BF_{10} > 10$, ** $BF_{10} > 30$, *** $BF_{10} > 100$.

Supplementary Table S4 Bayesian Pearson Correlations (9-10 group)

**Variable**		**RCPM**	**AS**	**VS**	**AVS**	**EHC**	**PPVT**	**EVT**	**RAN**
1. RCPM	Pearson's r	—							
	BF_{10}	—							
2. AS	Pearson's r	-0.403	—						
	BF_{10}	2.154	—						
3. VS	Pearson's r	-0.588 **	0.877 ***	—					
	BF_{10}	48.664	2.286e+7	—					
4. AVS	Pearson's r	-0.505	0.771 ***	0.862 ***	—				
	BF_{10}	9.439	19724.413	6.425e+6	—				
5. EHC	Pearson's r	-0.452	0.538	0.437	0.401	—			
	BF_{10}	2.589	8.147	2.170	1.507	—			
6. PPVT	Pearson's r	0.357	-0.092	-0.192	-0.182	-0.355	—		
	BF_{10}	0.968	0.281	0.372	0.359	0.833	—		
7. EVT	Pearson's r	0.572	-0.679 **	-0.570	-0.557	-0.540	0.695 **	—	
	BF_{10}	7.038	42.875	6.902	5.773	4.008	60.628	—	
8. RAN	Pearson's r	-0.341	0.327	0.346	0.337	0.378	-0.198	-0.396	—
	BF_{10}	1.012	0.895	1.058	0.973	1.216	0.382	1.121	—

Note. Age = age in numbers; RCPM = nonverbal IQ of Raven; AS = MRTs of auditory stimuli; VS = MRTs of visual stimuli; AVS = MRTs of audiovisual stimuli; EHC= Slurp visual motor skills; PPVT= Peabody picture vocabulary test; EVT= expressive vocabulary test; RAN = rapid automatized task. * $BF_{10} > 10$, ** $BF_{10} > 30$, *** $BF_{10} > 100$.

Supplementary Table S5 Bayesian Multiple Regressions for Age, Nonverbal IQ and Multisensory MRT (Auditory, visual, and audiovisual), EHC SLURP and RAN Predict Receptive and Expressive Vocabulary Tests

Model predictors	P(M)	P(M|data)	BF_M	BF_{10}	R^2
		***a.* PPVT**			
**Age + RCPM**	0.008	0.137	20.236	1.000	0.476
**Age**	0.008	0.098	13.844	0.715	0.430
**RCPM**	0.008	0.047	6.252	0.341	0.412
**Age + RCPM + AVS**	0.008	0.043	5.676	0.311	0.483
**Age + IQ + AS**	0.008	0.041	5.453	0.300	0.483
**Age + RCPM + EHC SLURP**	0.008	0.036	4.714	0.260	0.479
**Age + RCPM + RAN**	0.008	0.033	4.320	0.239	0.477
**Age + RCPM + VS**	0.008	0.032	4.140	0.230	0.476
**RCPM + VS**	0.008	0.027	3.589	0.200	0.438
**Age + AS**	0.008	0.027	3.564	0.199	0.438
		b. EVT			
**RCPM + AS**	0.008	0.095	13.316	1.000	0.665
**Age + RCPM**	0.008	0.080	10.991	0.839	0.662
**Age+ RCPM + AS**	0.008	0.075	10.250	0.787	0.688
**RCPM + AS + RAN**	0.008	0.053	7.160	0.562	0.683
**RCPM + AVS**	0.008	0.053	7.042	0.554	0.656
**RCPM + VS**	0.008	0.038	5.070	0.405	0.651
**Age + RCPM + AVS**	0.008	0.037	4.897	0.391	0.678
**RCPM + AVS + RAN**	0.008	0.035	4.612	0.369	0.677
**Age + RCPM + VS**	0.008	0.030	3.994	0.321	0.675
**Age + RCPM + RAN**	0.008	0.030	3.942	0.317	0.675

Note. Age = age in numbers; RCPM = nonverbal IQ of Raven; AS = MRTs of auditory stimuli; VS = MRTs of visual stimuli; AVS = MRTs of audiovisual stimuli; EHC= Slurp visual motor skills; PPVT= Peabody picture vocabulary test; EVT= expressive vocabulary test; RAN = rapid automatized task. .

Supplementary Table S6 Posterior Summaries of Regression Coefficients

Coefficient	P(incl)	P(incl|data)	BF$_{inclusion}$	Mean	SD	95% Credible Interval	
						Lower	Upper
		a. PPVT					
Intercept	1.000	1.000	1.000	138.113	2.370	133.871	143.015
**Agenumbers**	**0.500**	**0.804**	**4.095**	**5.660**	**3.849**	**-0.067**	**11.791**
**RCPM**	**0.500**	**0.685**	**2.175**	**0.982**	**0.884**	**0.000**	**2.575**
AS	0.500	0.241	0.318	0.003	0.013	-0.005	0.050
VS	0.500	0.263	0.358	-0.006	0.019	-0.055	0.027
AVS	0.500	0.254	0.341	0.005	0.020	-0.024	0.069
EHC SLURP	0.500	0.219	0.280	0.009	0.074	-0.134	0.195
RAN	0.500	0.209	0.264	0.006	0.118	-0.238	0.358
		b. *EVT*					
Intercept	1.000	1.000	1.000	100.373	1.519	97.448	103.568
Agenumbers	0.500	0.456	0.840	1.595	2.229	-0.367	6.091
**RCPM**	**0.500**	**0.985**	**64.072**	**1.535**	**0.459**	**0.729**	**2.552**
AS	0.500	0.470	0.886	-0.013	0.017	-0.050	0.000
VS	0.500	0.287	0.402	-0.007	0.017	-0.056	1.646e-4
AVS	0.500	0.304	0.436	-0.008	0.018	-0.061	0.000
EHC SLURP	0.500	0.207	0.261	-0.016	0.058	-0.238	1.731e-4
RAN	0.500	0.338	0.511	-0.079	0.145	-0.458	0.030

Note. Age = age in numbers; RCPM = nonverbal IQ of Raven; AS = MRTs of auditory stimuli; VS = MRTs of visual stimuli; AVS = MRTs of audiovisual stimuli; EHC= Slurp visual motor skills; PPVT= Peabody picture vocabulary test; EVT= expressive vocabulary test; RAN = rapid automatized task.

Supplementary Table S7 Bayesian Multiple Regressions for Age, Nonverbal IQ, PPVT, EVT and Rapid Automatized Naming (RAN) Predict Multisensory MRT (Auditory, visual, and audiovisual) and EHC SLURP

Model predictors	P(M)	P(M|data)	BF_M	BF_{10}	R^2
		a. AS			
**PPVT + EVT + Age**	0.031	0.273	11.633	1.000	0.453
**Age**	0.031	0.123	4.366	0.452	0.361
**EVT + Age**	0.031	0.100	3.449	0.367	0.395
**PPVT + EVT + Age + RCPM**	0.031	0.098	3.364	0.359	0.460
**PPVT + EVT + RAN + Age**	0.031	0.077	2.600	0.284	0.454
**EVT**	0.031	0.038	1.234	0.140	0.330
**PPVT + EVT**	0.031	0.034	1.082	0.124	0.367
**PPVT + EVT + RAN + Age + RCPM**	0.031	0.032	1.030	0.118	0.461
**PPVT + Age**	0.031	0.032	1.017	0.116	0.366
**EVT + Age + RCPM**	0.031	0.031	1.002	0.115	0.400
		b. VS			
**Age + RCPM**	0.031	0.195	7.494	1.000	0.532
**Age**	0.031	0.194	7.479	0.998	0.497
**EVT + Age**	0.031	0.164	6.075	0.842	0.528
**EVT + Age + RCPM**	0.031	0.080	2.684	0.409	0.545
**EVT + RAN + Age**	0.031	0.048	1.579	0.249	0.536
**RAN + Age + RCPM**	0.031	0.044	1.415	0.224	0.534
**PPVT + Age**	0.031	0.042	1.352	0.215	0.502
**PPVT + Age + RCPM**	0.031	0.041	1.338	0.213	0.533
**PPVT + EVT + Age**	0.031	0.038	1.229	0.196	0.531
**RAN + Age**	0.031	0.038	1.223	0.195	0.500
		c. AVS			
**Age**	0.031	0.263	11.089	1.000	0.482
**PPVT + EVT + Age**	0.031	0.134	4.793	0.508	0.535
**EVT + Age**	0.031	0.126	4.483	0.480	0.503
**Age + RCPM**	0.031	0.103	3.566	0.392	0.498
**PPVT + Age**	0.031	0.051	1.660	0.193	0.484
**RAN + Age**	0.031	0.050	1.616	0.188	0.483
**PPVT + EVT + RAN + Age**	0.031	0.049	1.591	0.185	0.543
**PPVT + EVT + Age + RCPM**	0.031	0.042	1.375	0.161	0.541
**EVT + Age + RCPM**	0.031	0.037	1.193	0.141	0.509
**EVT + RAN + Age**	0.031	0.033	1.073	0.127	0.507
		d. EHC			
**Age**	0.031	0.349	16.624	1.000	0.418
**RAN + Age**	0.031	0.149	5.439	0.428	0.438
**EVT + Age**	0.031	0.085	2.879	0.243	0.423
**Age + RCPM**	0.031	0.077	2.569	0.219	0.420
**PPVT + Age**	0.031	0.072	2.410	0.207	0.419
**EVT + RAN + Age**	0.031	0.040	1.306	0.116	0.440
**RAN + Age + RCPM**	0.031	0.040	1.301	0.115	0.440
**PPVT + RAN + Age**	0.031	0.038	1.219	0.108	0.438
**PPVT + EVT + Age**	0.031	0.028	0.905	0.081	0.430
**EVT + Age + RCPM**	0.031	0.022	0.697	0.063	0.423

Note. Age = age in numbers; RCPM = nonverbal IQ of Raven; AS = MRTs of auditory stimuli; VS = MRTs of visual stimuli; AVS = MRTs of audiovisual stimuli; EHC= Slurp visual motor skills; PPVT= Peabody picture vocabulary test; EVT= expressive vocabulary test; RAN = rapid automatized task.

Supplementary Table S8 Posterior Summaries of Regression Coefficients

**Coefficient**	**P(incl)**	**P(incl|data)**	**BF$_{inclusion}$**	**Mean**	**SD**	**95% Credible Interval**	
						Lower	Upper
			a. AS				
Intercept	1.000	1.000	1.000	870.743	16.020	841.710	903.974
**PPVT**	**0.500**	**0.589**	**1.433**	**1.369**	**1.501**	**-0.077**	**4.411**
**EVT**	**0.500**	**0.763**	**3.220**	**-3.182**	**2.432**	**-7.387**	**0.000**
RAN	0.500	0.218	0.278	-0.008	0.844	-2.143	2.341
**Age**	**0.500**	**0.883**	**7.523**	**-39.859**	**21.397**	**-71.086**	**0.000**
RCPM	0.500	0.240	0.316	0.447	2.194	-2.407	7.833
			b. VS				
Intercept	1.000	1.000	1.000	904.920	12.908	880.460	932.501
PPVT	0.500	0.191	0.237	-0.003	0.430	-1.335	0.754
EVT	0.500	0.421	0.728	-0.830	1.273	-3.933	0.003
RAN	0.500	0.202	0.253	-0.182	0.743	-2.210	0.909
**Age**	**0.500**	**0.972**	**34.109**	**-46.625**	**16.314**	**-82.156**	**-14.930**
RCPM	0.500	0.444	0.797	-2.390	3.438	-10.786	0.035
			c. AVS				
Intercept	1.000	1.000	1.000	824.625	12.366	799.090	846.770
PPVT	0.500	0.345	0.526	0.446	0.859	-0.146	2.540
EVT	0.500	0.459	0.849	-1.034	1.478	-4.104	0.304
RAN	0.500	0.204	0.257	-0.166	0.706	-2.238	0.385
**Age**	**0.500**	**0.989**	**88.537**	**-48.664**	**13.753**	**-73.538**	**-23.345**
RCPM	0.500	0.276	0.382	-0.919	2.213	-7.728	0.726
			d. EHC				
Intercept	1.000	1.000	1.000	67.373	2.070	63.472	72.073
PPVT	0.500	0.205	0.259	0.009	0.063	-0.101	0.195
EVT	0.500	0.231	0.300	-0.030	0.111	-0.386	0.133
RAN	0.500	0.327	0.485	0.087	0.177	-0.051	0.517
**Age**	**0.500**	**0.973**	**36.159**	**-6.671**	**2.123**	**-11.555**	**-2.638**
RCPM	0.500	0.214	0.271	-0.055	0.295	-1.002	0.402

Note. Age = age in numbers; RCPM = nonverbal IQ of Raven; AS = MRTs of auditory stimuli; VS = MRTs of visual stimuli; AVS = MRTs of audiovisual stimuli; EHC= Slurp visual motor skills; PPVT= Peabody picture vocabulary test; EVT= expressive vocabulary test; RAN = rapid automatized task.
